# Proteomic analysis of the excretory/secretory products and antigenic proteins of *Echinococcus granulosus* adult worms from infected dogs

**DOI:** 10.1186/s12917-015-0423-8

**Published:** 2015-05-21

**Authors:** Ying Wang, Di Xiao, Yujuan Shen, Xiuming Han, Fei Zhao, Xiaohong Li, Weiping Wu, Hejun Zhou, Jianzhong Zhang, Jianping Cao

**Affiliations:** National Institute of Parasitic Diseases, Chinese Center for Disease Control and Prevention; Laboratory of Parasite and Vector Biology, MOH, China; WHO Collaborating Center for Malaria, Schistosomiasis and Filariasis, Shanghai, 200025 China; Collaborative Innovation Center for Diagnosis and Treatment of Infectious Diseases, State Key Laboratory for Infectious Disease Prevention and Control, National Institute for Communicable Disease Control and Prevention, Chinese Center for Disease Control and Prevention, Beijing, 102206 China; Qinghai Institute for Endemic Disease Prevention and Control, Xining, 811602 China

**Keywords:** *Echinococcus granulosus*, Adult worm, Excretory/secretory products, Antigenic protein, 2-dimensional gel electrophoresis, MALDI-TOF/TOF

## Abstract

**Background:**

Cystic echinococcosis, which is caused by *Echinococcus granulosus*, is one of the most widespread zoonotic helminth diseases that affects humans and livestock. Dogs, which harbor adult worms in their small intestines, are a pivotal source of *E. granulosus* infection in humans and domestic animals. Therefore, novel molecular approaches for the prevention and diagnosis of this parasite infection in dogs need to be developed.

**Results:**

In this study, we performed proteomic analysis to identify excretory/secretory products (ES) and antigenic proteins of *E. granulosus* adult worms using two-dimensional electrophoresis, tandem matrix-assisted laser desorption/ionization time-of-flight (MALDI-TOF/TOF), and Western blotting of sera from infected dogs. This study identified 33 ES product spots corresponding to 9 different proteins and 21 antigenic protein spots corresponding to 13 different proteins. Six antigenic proteins were identified for the first time.

**Conclusions:**

The present study extended the existing proteomic data of *E. granulosus* and provides further information regarding host-parasite interactions and survival mechanisms. The results of this study contribute to vaccination and immunodiagnoses for *E. granulosus* infections.

## Background

Cystic echinococcosis (CE) is a type of zoonosis caused by *Echinococcus granulosus*, a canine tapeworm [1]. According to recent estimates, 4 million individuals are affected with CE and 40 million individuals are at risk [2–4]. The life cycle of *E. granulosus* is complex and involves two hosts: definitive and intermediate hosts. The definitive hosts are primarily dogs, which harbor adult worms in their small intestines. The intermediate hosts, e.g., humans and herbivores, particularly sheep and cattle, get infected through the ingestion of parasite eggs released in the feces of definitive hosts or through consumption of foods contaminated with the parasite eggs [5]. Dogs, as definitive hosts, are therefore pivotal in the transmission of CE.

One of the strategies to reduce the risk of infection is to interrupt the transmission of CE. Vaccination of the definitive hosts is an effective method. As there are far fewer dogs than sheep and cattle in endemic areas, far fewer animals consequently need to be vaccinated. However, a limitation of vaccination is that the immune modulatory mechanisms of *E. granulosus* are not fully understood. The immune modulatory mechanisms of the parasite might involve antigenic proteins and excretory-secretory (ES) products released by the parasite [6, 7]. The surveillance of *E. granulosus* infections in definitive hosts through sensitive diagnostic procedures is of paramount importance; coproantigen detection and arecoline purgation are not sufficiently sensitive. Therefore, there is an urgent need to identify immune markers that can be used in diagnosis and vaccine development. The identification of ES products and antigenic proteins could provide valuable insights into host-parasite interactions and improve the repertoire of candidate proteins used in immunodiagnoses, vaccination, and therapy. However, *E. granulosus* adult worms have received little attention; most studies have focused on the pre-adult stage of the parasite [8–11]. The proteomic profile of ES products and antigenic proteins from adult *E. granulosus* remain to be elucidated.

In this study, we investigated the ES products and antigenic proteins of *E. granulosus* adult worms from infected dogs using two-dimensional polyacrylamide gel electrophoresis (2DE) and tandem matrix-assisted laser desorption/ionization time-of-flight (MALDI-TOF/TOF) mass spectroscopy. The results obtained from this study are crucial for understanding the survival mechanisms of *E. granulosus* and host-parasite interactions. Furthermore, the results could assist in the development of vaccine antigens, drug targets, and immunodiagnosis markers.

## Methods

### Ethics statement

This study was performd in strict accordance with the recommendations of the Guide for the Care and Use of Laboratory Animals of the National Institute of Parasitic Diseases, Chinese Center for Disease Control and Prevention. The protocol was approved by the Laboratory Animal Welfare & Ethics Committee (LAWEC), National Institute of Parasitic Diseases, Chinese Center for Disease Control and Prevention (Permit Number: IPD 2010–007). Three dogs used in the research belonged to local farmers. The owners of the all dogs have oral consent for the use of their dogs in this research by explaining the purpose of the research and ensuring the welfare of animals.

### Collection and culture of parasites

All dogs were infected with *E. granulosus* protoscoleces from sheep after dewormed with praziquantel, and sacrificed to obtain intestines at 40 d post-infection. All surgery was performed under sodium pentobarbital anesthesia, and all efforts were made to minimize suffering. Worms were released by soaking the intestinal contents in phosphate-buffered saline (PBS, Gibco, California, USA), washed in sterile PBS containing 100 U/ml penicillin G and 100 μg/ml streptomycin (Gibco), and cultured for 24 h at 37 °C at 500 worms/ml in serum-free RPMI 1640 medium (Gibco) supplemented with 2 % glucose (Sigma, St.Louis, USA) and antibiotics. The supernatant was harvested and concentrated using a micro-concentrator with a 3-kDa cut-off (Millipore, Massachusetts, USA).

### Sample preparation

The concentrated supernatant was precipitated overnight at −20 °C with five volumes of ice-cold acetone containing 0.2 % dithiothreitol (DTT; w/v) and 20 % trichloroacetic acid (TCA; w/v). Protein precipitates were collected by centrifugation (10,000 rpm, 4 °C, 10 min) and washed three times with ice-cold acetone containing 0.2 % DTT (w/v). The resulting pellet was freeze-dried, suspended in lysate buffer (6 M urea, 2 M thiourea, 4.0 % CHARPS, 40 mM DTT, and 0.5 % IPG; pH 3–10), and sonicated in ice until the suspension became clear. The homogenate was centrifuged at 12,000 rpm for 15 min at 4 °C. The resulting supernatant contained ES products.

*E. granulosus* adult worms were washed in PBS, suspended in lysate buffer, and sonicated in ice until the suspension became clear. The homogenate was centrifuged at 12,000 rpm at 4 °C for 15 min. The supernatant contained adult worms.

The samples were cleaned up using the 2D Clean-up kit (Amersham Biosciences), quantified using the 2D Quant Kit (Amersham Biosciences), and subjected to 2DE.

### Two-dimensional electrophoresis

One-dimensional isoelectric focusing (IEF) and two-dimensional sodium dodecyl sulfate polyacrylamide gel electrophoresis (SDS-PAGE) were performed (Amersham Biosciences). Briefly, 100 μg of the ES sample in 130 μl rehydration buffer was loaded onto a 7-cm immobiline IPG drystrip (pH3-10NL; Amersham Biosciences); 800 μg of adult worm protein sample in 450 μl of rehydration buffer was loaded onto a 24-cm immobiline IPG drystrip (pH3-10NL; Amersham Biosciences). IEF was performed at 80,000 Vh using Ettan™IPGphorII (Amersham Biosciences). The drystrips were equilibrated twice (15 min each time) in equilibration buffer (50 mM Tris–HCl [pH 8.8], 6 M urea, 30 % glycerol, 2 % SDS, and 1 % DTT for the first equilibration; 50 mM Tris–HCl [pH 8.8], 6 M urea, 30 % glycerol, 2 % SDS, and 4.8 % iodacetamide for the second equilibration). Two-dimensional SDS-PAGE was performed on a 12 % polyacrylamide gel in the Ettan™DALTsix and Ettan™VE systems (Amersham Biosciences). Two replicates were performed per protein sample.

### Protein identification by MALDI-TOF/TOF analysis

The gels were stained with Coomassie brilliant blue (CBB) G-250 (BioRad). Protein spots were excised by an Ettan Spot Picker (Amersham Biosciences). In-gel digestion was performed as previously described [12]. Protein identification was carried out using MALDI-TOF/TOF mass spectrometry (4700 MALDI-TOF/TOF mass spectrometer; Applied Biosystems, California, USA). The spectra were processed and analyzed by the Global Protein Server Workstation (GPS; Applied Biosystems), which uses the internal Mascot v2.1 software (Matrix Science, London, UK) to search for peptide mass fingerprints and MS/MS data based on the NCBI non-redundant protein database. Identification with a GPS confidence interval >95 % was accepted. Gene ontology (GO) terms were applied to the identified proteins; pie charts of the GO terms for molecular functions, cellular components, and biological processes were generated. Additionally, Eukaryotic Orthologous Group (KOG) annotation was assigned (http://genome.jgi.doe.gov/Tutorial/tutorial/kog.html).

### Immunoblot analysis of antigenic proteins

Adult worm proteins were electro-transferred from the 2DE gels to polyvinylidene fluoride (PVDF) membranes (Amersham Biosciences) at 300 mA for 2 h using a TE77 semi-dry transfer unit (Amersham Biosciences). Membranes were blocked for 1 h in 5 % nonfat milk powder in TBS (20 mM Tris, 500 mM NaCl, pH 7.5) at room temperature and incubated overnight with serum from *E. granulosus*-infected dogs (1:500 dilution) at 4 °C. After three washes with TBST (TBS containing 0.05 % v/v Tween-20), the membranes were incubated at room temperature with HRP-labeled anti-dog IgG (1:10000 dilution; Sigma-Aldrich) for 1 h. Bound antibodies were revealed using the DAB reagent. Antigenic spots on the 2DE gels were identified based on matches with the 2DE proteomic map.

## Results

### ES products of adult *E. granulosus*

**Fig. 1:**
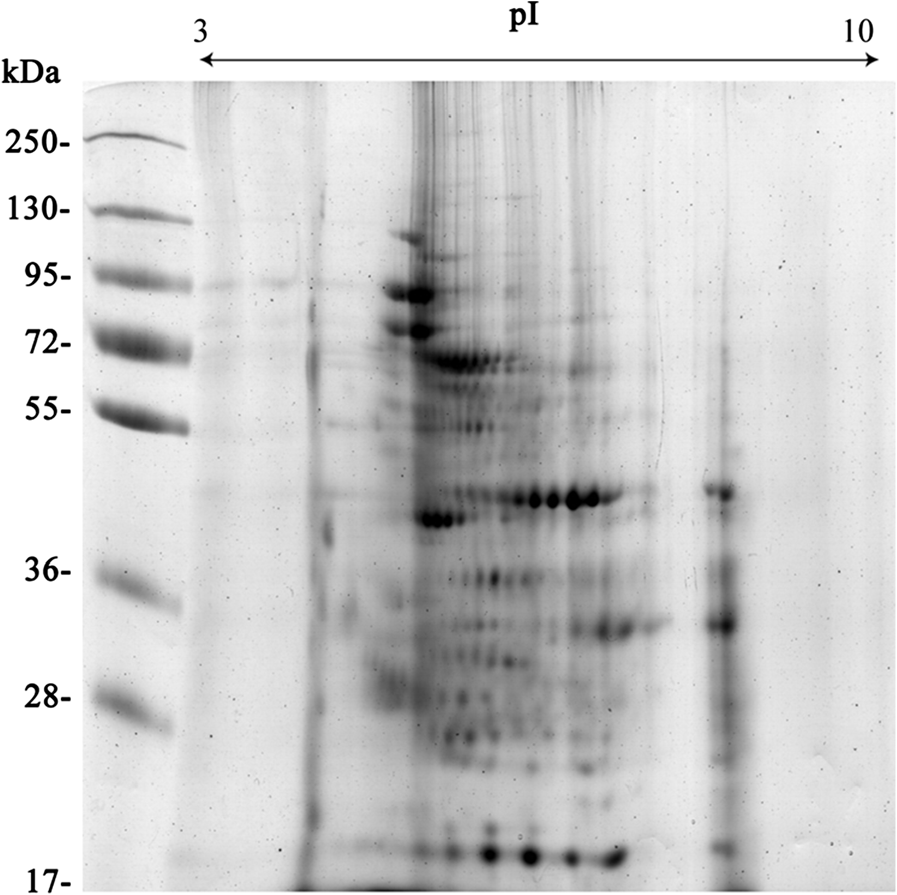
Representative 2DE gels of the excretory/secretory products of *E. granulosus* adult worms. Proteins (100 μg) were separated on a linear pH range of 3–10 using IEF in the first dimension and 12 % SDS-PAGE in the second dimension. The proteins were stained with Coomassie *blue*. Molecular weight markers are shown on the *left*. The proteins identified are shown in detail in Table 1

Approximately 50 spots representative of the ES products were resolved on Coomassie-stained 2DE gels (Fig. 1). A total of 48 spots were subjected to MALDI-TOF/TOF analysis; 33 protein spots corresponding to 8 different proteins were identified (Table 1).

**Table 1:** ES products of *E. granulosus* adult worms identified by MALDI-TOF/TOF analysis

GI number	Protein name	Species	MW	p*I*	Protein score C.I.%
547974	Paramyosin	*E. granulosus*	98681.8	5.21	100
262192839	Enolase	*E. granulosus*	46531.9	6.48	100
576695773	Actin	*E. granulosus*	41717.8	5.3	100
576698524	Small heat shock protein p36	*E. granulosus*	35821.2	5.92	99.995
6016537	Malate dehydrogenase, cytoplasmic	*E. granulosus*	36627.8	8.11	100
2316076	Glutathione S-transferase	*E. granulosus*	25536.8	7.51	100
189016336	Thioredoxin peroxidase	*E. granulosus*	21419.7	5.78	100
31077167	Cyclophilin	*E. granulosus*	17343.4	6.41	99.003

**Fig. 2:**
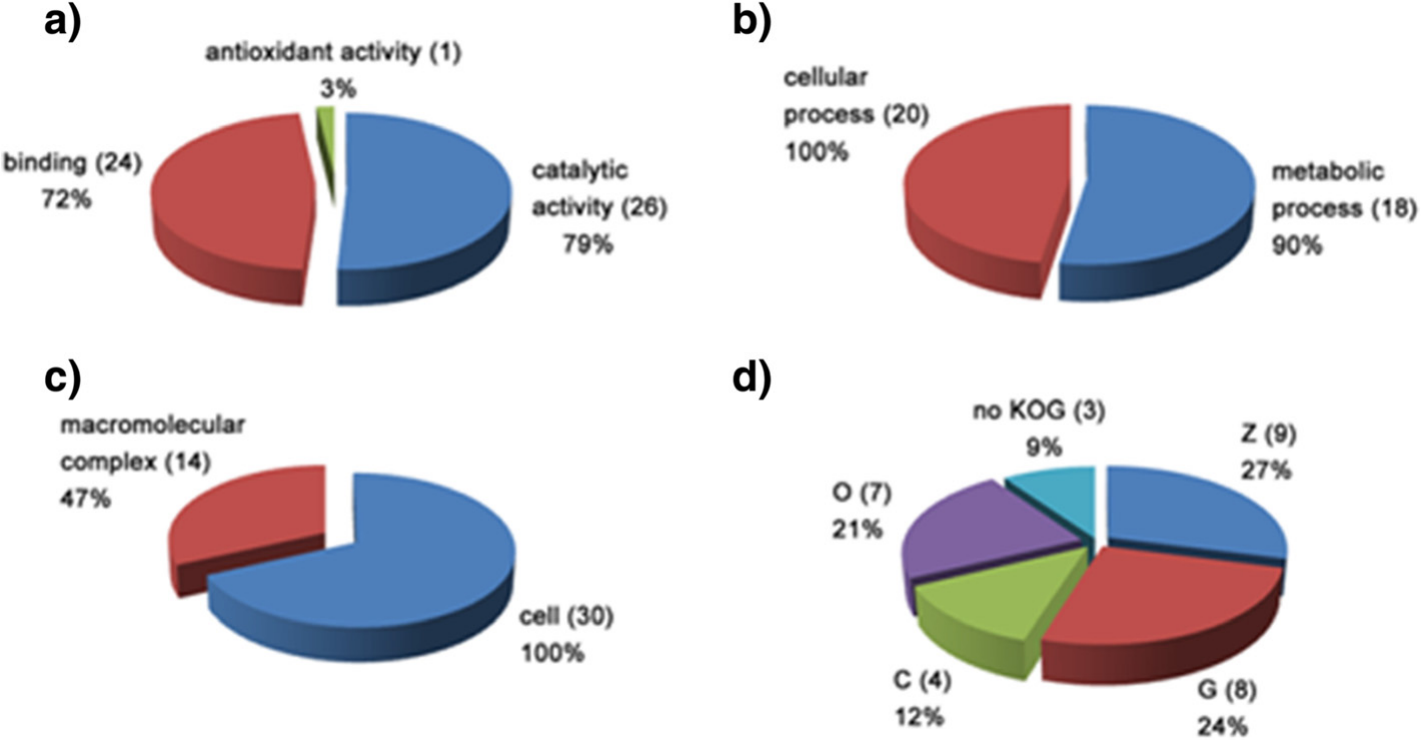
Functional analysis of the excretory/secretory proteins identified in *E. granulosus* adult worms. Gene ontology terms for the subcategories molecular function (**a**), cellular component (**b**), and biological process (**c**) were assigned to the proteins identified in the adult worms. KOG functional categories (**d**) were assigned to the proteins identified in the adult worms. The percentage and number (in parentheses) of proteins identified in each functional category are indicated in each sector. KOG functional categories: (Z) cytoskeleton; (O) posttranslational modification, protein turnover, chaperones; (G) carbohydrate transport and metabolism; (C) energy production and conversion; (noKOG) protein not assigned to any KOG category. The number of proteins in the graph might exceed the total number of identified proteins because some were grouped into more than one functional category

The identified proteins were subjected to functional annotation based on the GO and KOG classification systems. 33, 30, and 20 proteins had GO terms for molecular functions, cellular components, and biological processes, respectively. A summary of the GO and KOG annotations is provided in Fig. 2. Three proteins could not be classified by KOG.

### Antigenic proteins of *E. granulosus* adult worms

**Fig. 3:**
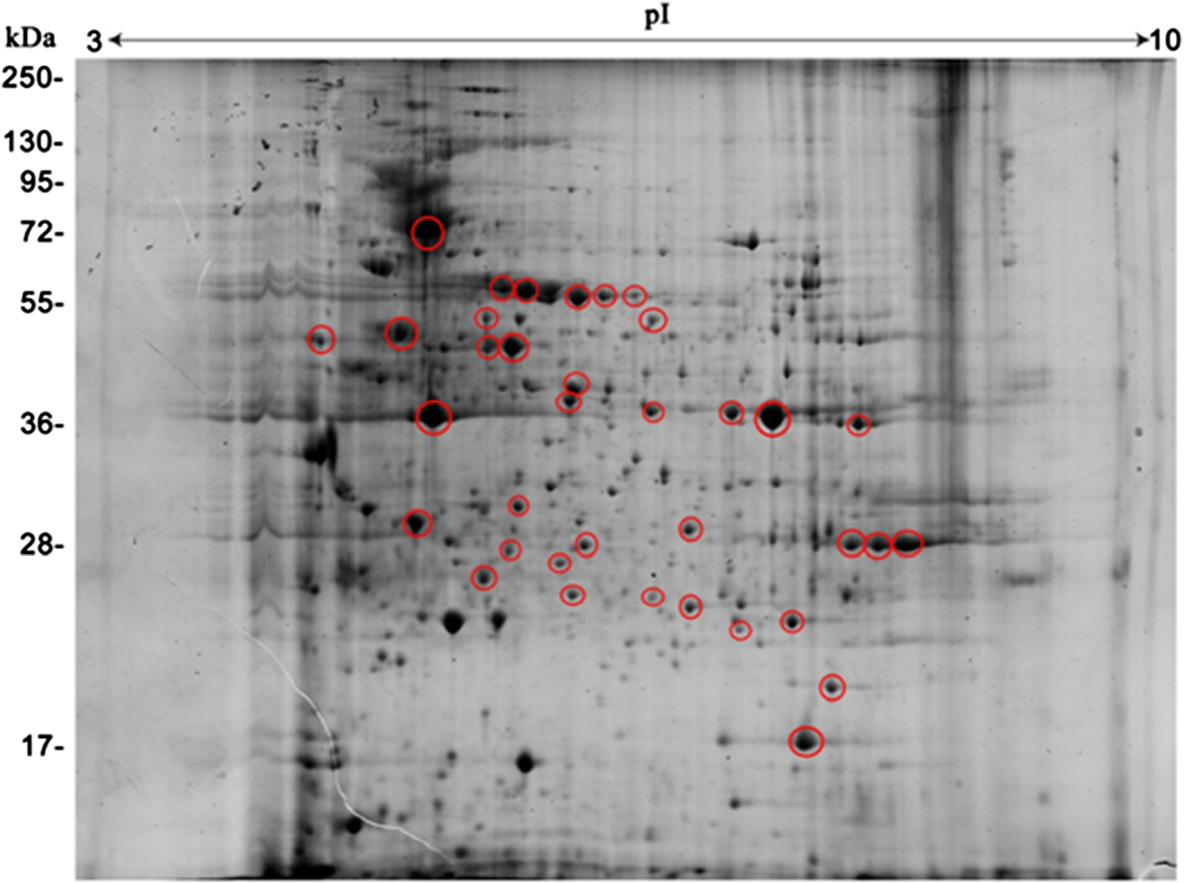
Antigenic proteins on corresponding representative 2DE gels of the proteins expressed by *E. granulosus* adult worms. Proteins (800 μg) were separated on a linear pH range of 3–10 by using IEF in the first dimension and 12 % SDS-PAGE in the second dimension. Proteins were electrotransferred to PVDF membranes and probed with sera from *E. granulosus*-infected dogs. Antigenic protein spots are indicated by red circles. Molecular weight markers are shown on the left. The proteins identified are shown in detail in Table 2

To identify adult worm antigenic proteins, we performed 2DE immunoblot analyses using serum from *E. granulosus*-infected dogs. In this study, 36 antigen spots were detected (Fig. 3). Using the corresponding 2DE proteomic map, 21 antigen spots were identified, corresponding to 12 different proteins (Table 2), three of which were identified for the first time: severin, hypothetical protein EGR_06319, and triosephosphate isomerase.

**Table 2:** Antigenic proteins of *E. granulosus* adult worms identified by immunoblot analysis

GI number	Protein name	Species	MW	p*I*	Protein score C.I.%
547974	Paramyosin	*E. granulosus*	98681.8	5.21	100
148613837	Calreticulin	*E. granulosus*	42199.2	4.47	100
256274460	HSP60	*E. granulosus*			
674568928	HSP70	*E. granulosus*	70640	5.47	100
26399708	**Severin** ^**a**^	*E. granulosus*	41681.9	5.74	100
262192839	Enolase	*E. granulosus*	46531.9	6.48	100
576695773	Actin	*E. granulosus*	41717.8	5.3	100
576695197	**Hypothetical protein EGR_06319** ^**a**^	*E. granulosus*	23119	4.77	100
6016537	Malate dehydrogenase, cytoplasmic	*E. granulosus*	36627.8	8.11	100
576692880	**Triosephosphate isomerase** ^**a**^	*E. granulosus*	27149.8	6.6	100
576691284	Superoxide dismutase	*E. granulosus*	15442.8	6.15	99.992
31077167	Cyclophilin	*E. granulosus*	17343.4	6.41	99.003

^a^Antigenic proteins identified for the first time in adult worms are shown in bold

A total of 61 adult worm protein spots corresponding to 26 different proteins were identified (Table 3); 2 of these proteins were identified in *E. granulosus* adult worms for the first time.

**Table 3:** Proteins of *E. granulosus* adult worms identified by MALDI-TOF/TOF analysis

GI number	Protein name	Species	MW	p*I*	Protein score C.I.%
547974	Paramyosin	*E. granulosus*	98681.8	5.21	100
148613837	Calreticulin	*E. granulosus*	42199.2	4.47	100
256274460	HSP60	*E. granulosus*			
674568928	HSP70	*E. granulosus*	70640	5.47	100
576695773	Actin	*E. granulosus*	41745.8	5.39	100
26399708	Severin	*E. granulosus*	41681.9	5.74	100
262192839	Enolase	*E. granulosus*	46531.9	6.48	100
576695773	**Actin** ^**a**^	*E. granulosus*	41717.8	5.3	100
576695197	Hypothetical protein EGR_06319	*E. granulosus*	23119	4.77	100
6016537	Malate dehydrogenase, cytoplasmic	*E. granulosus*	36627.8	8.11	100
576692880	Triosephosphate isomerase	*E. granulosus*	27149.8	6.6	100
576691284	Superoxide dismutase	*E. granulosus*	15442.8	6.15	99.992
31077167	Cyclophilin	*E. granulosus*	17343.4	6.41	99.003
29336624	78-kDa glucose-regulated protein	*E. granulosus*	71876.1	5.16	100
674566315	Transitional endoplasmic reticulum atpase	*E. granulosus*	71581.6	5.17	100
110558962	Ferritin	*E. granulosus*	16676.2	5.24	100
189016336	Thioredoxin peroxidase	*E. granulosus*	21419.7	5.78	100
674565853	Succinate dehydrogenase ubiquinone	*E. granulosus*	54812.5	7.62	100
576691476	Transketolase	*E. granulosus*	72574.6	6.53	100
576693013	**T-complex protein 1 subunit zeta** ^**a**^	*E. granulosus*	26703	7.77	100
182676451	Tropomyosin	*E. granulosus*	32249.2	4.6	100
576698524	Small heat shock protein p36	*E. granulosus*	35821.2	5.92	99.995
674567794	Oncosphere protein Tso22e	*E. granulosus*	31416.3	6.61	100
2316076	Glutathione S-transferase	*E. granulosus*	25536.8	7.51	100
193213138	Phosphoglycerate mutase	*Chlorobaculum parvum*	28366.6	5.76	95.745
576700988	Fructose-bisphosphate aldolase	*E. granulosus*	39702.4	8.03	100

^a^Proteins identified for the first time in adult worms are shown in bold

## Discussion

In this study, we performed the first proteomic analysis of ES products from adult *E. granulosus*. Enolase was the most abundant ES product Enolase has been described as a multifunctional surface protein and an ES product of parasites; it exhibits host-interacting properties and is involved in invasion [13–17]. Enolase is likely to play an important role in *E. granulosus*-host interactions and parasite evasion mechanisms *via* the immunomodulation of the host immune system. The second most abundant ES product was cyclophilin, which plays a role in parasite development and host-parasite interactions [18]. Furthermore, cyclophilin is a target compound for the immunosuppressive agent, cyclosporine A [19–21].

Actin and paramyosin, cytoskeletal proteins, were other ES products. Similar results have been obtained in other parasites [22–25]. The presence of these proteins may indicate parasite damage or death; however, it is more likely that these proteins are products of tegument shedding. The continuous shedding of the parasite tegument is thought to release components that aid the parasite in evading an effective immune response [26].

An antioxidant defense mechanism is another form of parasite survival. Among the ES products identified, only thioredoxin peroxidase was involved in redox homeostasis. This enzyme plays a major role in protecting the adult worms from oxidative damage. This result is in accordance with the findings of a study performed of hydatid cyst fluid (HCF) that contained ES products from *E. granulosus* protoscoleces [9].

Metacestodes (hydatid cysts), protoscoleces, and adult worms are different developmental stages of *E. granulosus*. The HCF is a reservoir of ES products from both the germinal layer and protoscoleces. Compared with HCF [9] and ES products from protoscoleces [11], the ES products from adult worms comprise different proteins. Some of the proteins in this study have not been identified in HCF, including enolase, malate dehydrogenase, GST, and cyclophilin. Similarly, some of the proteins have not been identified in the ES products from protoscoleces, including paramyosin, small HSP p36, and GST. In another study, we observed that the ES products from *E. granulosus* adult worms failed to induce dendritic cell maturation and cytokine production (data not published); this result, however, is in contrast with the finding that HCF induces dendritic cell activation [27]. These differences could be attributed to the different proteins in ES products and HCF; however, this needs to be confirmed.

The search for antigenic proteins by 2DE immunoblots contributed to the identification of 12 parasite proteins that were recognized in the serum of *E. granulosus*-infected dogs. Nine of these proteins (malate dehydrogenase, HSP60, HSP70, calreticulin, enolase, actin, cyclophilin, paramyosin and superoxide dismutase) were previously identified in *E. granulosus* and *E. multilocularis* human infections [9, 18, 28–30]. The other three proteins (severin, hypothetical protein EGR_06319 and triosephosphate isomerase,) were identified in this study for the first time. Some of these proteins have potential in vaccine development, such as paramyosin. Paramyosins, which are multifunctional modulators of the host immune response, play a role in binding complement components, immunoglobulins, and secreted components of cellular immune response [31–36]. Paramyosin has been proposed by WHO/TDR as a vaccine candidate against schistosomiasis and has shown some protection, which may suggest a potential for this protein in the treatment of *E. granulosus* infections [37–39].

Recently, the protein profile of *E. granulosus* adult worms has been analyzed by two dimensional LC-MS [40]. In addition to proteins that had been reported in that work, we identified two novel proteins of this parasite, which has increased the known repertoire of the parasitic protein profile and demonstrates the value of our experimental approaches for the proteomic analysis of *E. granulosus* proteins.

## Conclusions

The present study investigated the ES products and antigenic proteins of *E. granulosus* adult worms from infected dogs. The results obtained from this study extend the existing proteomic data regarding *E. granulosus* and could assist in the development of vaccine antigens, drug targets, and immunodiagnostic markers.

## Abbreviations

2DE: Ttwo-dimensional polyacrylamide gel electrophoresis
CE: Cystic echinococcosis
ES: Excretory-secretory products
GO: Gene ontology
KOG: Eukaryotic Orthologous Group (KOG)
MALDI-TOF/TOF: Tandem matrix-assisted laser desorption/ionization time-of-flight
